# Findings and outcomes of hospitalized unvaccinated patients during the COVID-19 pandemic: impact of comorbidities on clinical, laboratory, and immunological parameters

**DOI:** 10.1590/S1678-9946202567059

**Published:** 2025-08-25

**Authors:** Georon Ferreira de Sousa, Jéssica Pires Farias, Bárbara Rafaela da Silva Barros, Danilo Bancalero Mendonça Lucchi, Simone Ravena Maia Alves, Guilherme Antonio da Souza Silva, Leonardo Carvalho de Oliveira Cruz, Rodrigo Cesar Abreu de Aquino, Edson Barbosa de Souza, Evonio de Barros Campelo, Antonio Carlos de Freitas, Luís Carlos de Souza Ferreira, Carla Torres Braconi, Cristiane Moutinho-Melo

**Affiliations:** 1 Universidade Federal de Pernambuco, Centro de Biociências, Laboratório de Análises Imunológicas e Antitumorais, Departamento de Antibióticos, Recife, Pernambuco, Brazil; 2 Universidade Federal de Pernambuco, Instituto Laboratório de Imunopatologia Keizo Asami, Recife, Pernambuco, Brazil; 3 Universidade de São Paulo, Instituto de Ciências Biomédicas, Departamento de Microbiologia, Laboratório de Desenvolvimento de Vacinas, São Paulo, São Paulo, Brazil; 4 Universidade Federal de São Paulo, Escola Paulista de Medicina, Departamento de Microbiologia, Imunologia e Parasitologia, São Paulo, São Paulo, Brazil; 5 Universidade Federal de Pernambuco, Hospital das Clínicas, Recife, Pernambuco, Brazil; 6 Universidade Federal de Pernambuco, Centro de Biociências, Departamento de Genética, Laboratório de Estudos Moleculares e Terapia Experimental, Recife, Pernambuco, Brazil; 7 Universidade de São Paulo, Plataforma Científica Pasteur USP, São Paulo, São Paulo, Brazil

**Keywords:** COVID-19, Comorbidities, Immune response, Inflammatory markers

## Abstract

The COVID-19 pandemic continues to highlight the significant impact of pre-existing comorbidities on disease progression and patient outcomes due to the risk factors for severe disease in unvaccinated patients. We evaluated the association between several clinical/laboratory findings and comorbidities in a cohort of unvaccinated patients hospitalized in the intensive care unit in Recife, Pernambuco State, Brazil. We enrolled 36 unvaccinated volunteers, and performed clinical, biochemical, hematological, and microbiological analyses. Cellular immunity, cytokine measurement, and gene expression were also analyzed. Additionally, serum samples were submitted to serological and neutralization assays by using SARS‐CoV‐2 B.1 Lineage, Gamma (P.1), Delta (B.1.617.2-like), and Omicron (BA.1) variants. Hypertension was the most common comorbidity in patients requiring oxygen supplementation, followed by diabetes and metabolic syndrome. Such conditions were linked to increased disease severity, with elevated levels of inflammatory biomarkers (D-dimer, C-reactive protein), neutrophilia, and lymphopenia. Chronic inflammation, which is often seen in diabetes and metabolic syndrome, worsens the inflammatory response triggered by COVID-19, which exacerbates endothelial injury and leads to a hypercoagulable state. Additionally, patients with comorbidities had impaired humoral immunity, and showed reduced seroconversion and neutralizing activity, which hindered their ability to combat the virus effectively. Furthermore, this study revealed that patients with diabetes and metabolic syndrome had an exaggerated Th17-driven immune response, which contributed to severe outcomes and multi-organ failure. These findings underscore the importance of personalized care and targeted interventions for patients with comorbidities, thus highlighting the need for further research on metabolic disorders, immune dysfunction, and COVID-19.

## INTRODUCTION

Almost five years after the COVID-19 (Coronavirus Disease 2019) pandemic onset, which has led to approximately 776 million cases and seven million deaths, the association between the consequences of pre-existing comorbidities and COVID-19, as well as their significance in patient prognosis**,** continues to be under investigation^1^. In April, the Centers for Disease Control and Prevention (CDC) compiled an updated list of medical conditions that were highly associated with an increased risk of severe COVID-19, including diabetes, hypertension, cardiovascular disease, and obesity^2^. The COVID-19 infection, *per* se, leads to an exacerbated immune response by inducing a cytokine storm, which is characterized by the excessive release of pro-inflammatory cytokines, such as interleukin-6 (IL-6) and tumor necrosis factor-alpha (TNF-alpha)^3^. This hyperactive immune response associated with these comorbidities plays an important role in precipitating severe respiratory distress, thromboembolic events, and multi-organ dysfunctions^4^.

Managing multiple health conditions poses a significant challenge to public health systems in Brazil, and studies on the relationship between comorbidities and COVID-19 in the Brazilian population remain unclear for several reasons. Firstly, despite vaccination efforts, variation in vaccination rates across different regions means that large portions of the population remain unvaccinated or under-vaccinated, which impacts disease dynamics and complicates comparative analyses^5^. Secondly, Brazil’s diverse socioeconomic landscape and healthcare disparities contribute to uneven access to healthcare services and data collection infrastructure, thus hindering comprehensive and representative studies^6,7^. The complexity of managing a diverse and multicultural population, together with logistical and infrastructural limitations, contributes to the ongoing difficulty in accurately tracking and addressing the intersection of comorbidities and COVID-19, mainly in the Northeast region of the country^8^. Additionally, the Brazilian population has distinct characteristics in the distribution of comorbidities, with a high rates of cardiovascular diseases and diabetes. Currently, more than 20 million people live with diabetes, and over 30% of the population has hypertension^9,10^. Understanding how these factors influence the progression of COVID-19 is essential for tailoring treatment approaches and public health policies to effectively meet the specific needs of the local population.

Herein, we aimed to evaluate the association between several clinical/laboratory findings and comorbidities among a cohort of patients hospitalized in the ward or intensive care unit (ICU) in Recife, Pernambuco State, Brazil, during the COVID-19 pandemic period. Clinical and laboratory data—including pre-existing conditions like hyperglycemia and hypertension—were analyzed in unvaccinated patients to better understand immune, biochemical, and physiological responses during the COVID-19 progression. This study was conducted during the pre-vaccination phase, which enabled a detailed investigation of immune markers, including helper T cells and exhausted lymphocytes. These conclusions are crucial for developing specific public health strategies, identifying high-risk groups, and refining protocols that meet the health needs of these vulnerable individuals.

## MATERIALS AND METHODS

### Study design and sample collection

**Figure 1:**
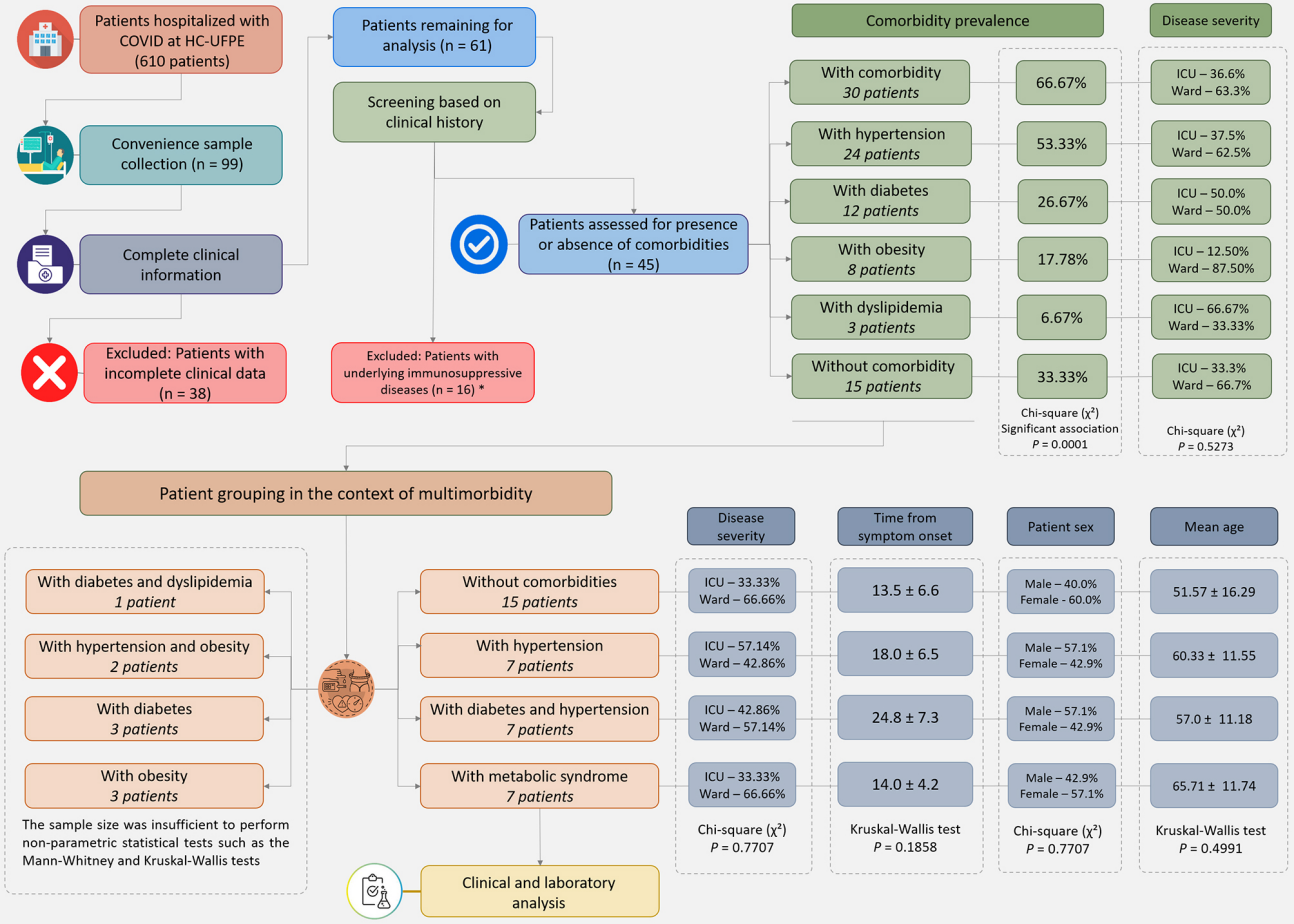
Study design: flowchart showing the stages of the study from patient selection to the formation of groups for clinical and laboratory analysis; *patients without comorbidities but with transient self-limiting conditions that could interfere with laboratory analyses were also excluded.

This is a retrospective observational study on a hospitalized population from Recife, Pernambuco State, Brazil. Data and samples were collected during the pre-vaccination period at the Clinic Hospital of the Federal University of Pernambuco from July 2020 to March 2021. During the study period, 610 patients were admitted to both the ward and the ICU with a confirmed positive COVID-19 diagnosis, as determined by RT-qPCR. We were only able to collect samples from 99 individuals due to biosafety measures. Of these, 36 were selected based on the criteria of having at least one comorbidity and complete clinical information in the medical records (Figure 1). Children and patients with other severe respiratory syndromes were excluded.

All the laboratory findings (biochemical, hematological, and microbiological analyses) were performed at the hospital’s laboratory department, in which serum, whole blood, and swab samples from tracheal, nasal, rectal regions, and urine were used. These clinical exams were conducted upon request from the hospital’s multidisciplinary team. Patient admission history, symptoms, treatments, imaging exams, comorbidities, co-infections, and other relevant clinical aspects were evaluated in the medical records. Additionally, we collected blood samples to obtain immunological profiles with ELISA and neutralization assays, including cell immunophenotyping, cytokine measurement, and gene expression analysis.

### Comorbidities and group classification

The types of comorbidities were evaluated based on medical records. To determine whether they had metabolic syndrome, we used the criteria defined by the National Cholesterol Education Program Adult Treatment Panel III (NCEP ATP III) as follows: elevated abdominal circumference, high triglyceride levels, low HDL cholesterol levels, elevated blood pressure, and high fasting-glucose levels^11^. Based on clinical analyses and the criteria established by NCEP ATP III^9^, the 36 patients were divided into different groups according to their type of comorbidities: Group 1 (G1) had patients without comorbidities (*n* = 15); Group 2 (G2) had patients with systemic arterial hypertension (SAH) (*n* = 7); Group 3 (G3) had patients with type 2 diabetes mellitus (DM) and SAH (*n* = 7); and Group 4 (G4) had patients with metabolic syndrome (MetS) (*n* = 7).

### Ethics

This study was approved by the local research ethics committee and all individuals signed an informed consent form under CAAE Nº 30332120.8.3001.5191 and Substantive Opinion of the Ethics Committee Nº 4.206.047. The enrolled patients did not receive COVID-19 vaccines.

### Blood cells count and biochemical analysis

Blood samples were obtained via venipuncture and collected in BD Vacutainer^®^ tubes (BD, USA). Hematological analysis was performed by using the Yumizen H2500^®^ hematology analyzer (HORIBA Medical, Japan) according to the manufacturer’s instructions, followed by optical microscopy for examination and description of any morphological changes and cellular inclusions. Other tests, such as coagulation time, prothrombin time (PT), activated partial thromboplastin time (APTT), international normalized ratio (INR), and fibrinogen levels, were analyzed by using the automatic STA Compact Max^®^ analyzer (Stago^®^, USA). Biochemical parameters such as urea, creatinine, creatine phosphokinase (CPK), lactate dehydrogenase (LDH), ferritin, gamma-glutamyl transferase (GGT), aspartate aminotransferase (AST), alanine aminotransferase (ALT), C-reactive protein (CRP), bilirubin, D-dimer, and alkaline phosphatase were determined by using the automated Architect i2000SR^®^ analyzer (Abbott^®^, USA).

### Microbiological analysis

The microbiological laboratory diagnosis was performed by using blood, swab samples from the tracheal, nasal, rectal regions, and urine. The clinical samples were immediately inoculated onto blood agar and MacConkey agar and incubated at 35-37 °C for 18 to 24 h under appropriate atmospheric conditions. After growth, microorganisms were identified with phenotypic and biochemical tests. Catalase test was performed on *Staphylococcus* spp. isolates, and their growth was evaluated on DNase agar to assess novobiocin resistance, thus aiding in the differentiation of *Staphylococcus saprophyticus*. Catalase-negative, alpha-hemolytic bacteria were seeded on bile-esculin and infusion agar, whereas catalase-negative, beta-hemolytic bacteria were tested for CAMP, bacitracin, and PYR. Bacteria isolated from MacConkey agar were identified by laboratory tests defined in the Clinical and Laboratory Standards Institute protocol (CLSI)^12^. For bacteria identified in manual testing, cultures were submitted to the automation system for confirmation of species and antimicrobial susceptibility by using BD Phoenix^®^ equipment. All reports were released based on the database of the Brazilian Committee for Antimicrobial Susceptibility Testing (BrCAST).

### Serological assays

#### Enzyme-linked immunosorbent assay (ELISA)

ELISA tests were performed with Wuhan RBD protein from SARS-CoV-2 (the plasmid encoding RBD was kindly provided by Dr. Florian Krammer [Icahn School of Medicine at Mount Sinai, USA]) to detect IgM and IgG antibodies in the patients’ serum^13^. Briefly, polystyrene microplates (96 wells) were coated with the protein overnight at 4 °C before being incubated with blocking solution (3% milk, 1% BSA in 0.05% PBS-Tween) at 37 °C for 2 h. Then, twofold diluted (1:20–1:2560), heat‐inactivated serum samples were added to the wells and incubated at 37 °C for 1 h. After the washing cycles, the samples were stained with peroxidase-conjugated anti-human IgM and IgG antibodies (Sigma-Aldrich^®^, USA) at 1:2000 and 1:4000 dilution, respectively , at 37 °C for 1 h. The revel color was obtained by OPD solution (Sigma-Aldrich^®^, USA) mixed with sodium citrate buffer (pH 5.8). After 15 min of incubation, the stop solution (2N - H_2_SO_4_) was added. The absorbance was measured at 492 nm with an Epoch^®^ spectrophotometer plate reader (Agilent Technologies^®^, USA). Antibodies titers were defined according to optical density values (OD). The cut-off value was defined as the mean of OD values of negative samples plus four times the standard deviation (SD) value, meaning a 20-percent cut-off threshold. All the samples with ODs above the threshold zone were considered positive.

#### Neutralization Assay

Cytopathic effect‐based virus neutralization test (CPE‐VNT) for SARS‐CoV‐2 B.1 lineage and VOCs: all experiments involving SARS-CoV-2 propagation, titration, and CPE-VNT were performed in a biological safety level 3 (BSL3) laboratory according to the World Health Organization (WHO) recommendations^14,15^. Briefly, cell monolayers (5×10^4^ Vero CCL‐81 ATCC cells/well) in 96‐well culture plates were exposed to 1×10^2^ TCID50/mL of SARS‐CoV‐2 B.1 Lineage (GISAID: EPI_ISL_412964), or variants of concern Gamma (P.1) (GISAID:EPI_ISL_1060981), Delta (B.1.617.2-like) (GISAID: EPI_ISL_2965577), and Omicron (BA.1) (GISAID: EPI_ISL_6901961),which were previously incubated with 1:20 to 1:1280 twofold diluted, heat‐inactivated human serum samples, yielding a final volume of 150 µL. After 72 h of incubation, the plates were microscopically evaluated for the characteristic cytopathic effects (CPEs) of SARS‐CoV‐2. The absence of CPEs in the 1:20 diluted sample was considered a positive result for the presence of neutralizing antibodies against SARS‐CoV‐2.

#### Isolation and culture of peripheral blood mononuclear cells (PBMC) and cell immunophenotyping

PBMCs were isolated by using a concentration gradient technique (Ficoll‐Paque^®^ Plus, GE Healthcare Life Science, Sweden). Blood samples were centrifuged (900x*g*/30 min/20 °C) and after separation of PBMCs, the cells were washed twice with sterile PBS (400×*g*/10min/20 °C). Lymphocyte immunophenotyping was performed by using CD3-FITC, CD4-PerCP.Cy5.5, CD8-PE, CD19-APC, CD56-PE.Cy7 antibodies. Data acquisition was performed with a FACS-VERSE flow cytometer (BD^®^, USA) in 100,000 events, and data analysis was performed by using the Flowing software (version 2.01, BD^®^, USA).

#### Serum Cytokine Measurement

The collected serum samples were submitted to cytokine investigation by using the CBA Th1/Th2/Th17 kit (BD^®^, USA), which enabled simultaneous detection of IL-2, IL-4, IL-6, IL-10, IL-17, TNF-α, and IFN-γ cytokines. The samples were processed according to the protocol recommended by the manufacturer. Data were acquired with a BD Accuri™ C6 cytometer (BD^®^, USA), with 2,100 events collected *per* sample, and the analysis was conducted with BD Accuri™ C6 Software Plus BD^®^.

## Gene expression analysis

### RNA extraction and cDNA synthesis

PBMCs of patients (5x10^6^) were homogenized with 1 mL of TRIzol (Invitrogen^®^, USA) for RNA extraction. Total RNA was purified by using the RNeasy^®^ Mini Kit (QIAGEN^®^, Germany) according to the manufacturer’s instructions and was measured with a NanoDrop 2000 spectrophotometer (Thermo Scientific^®^, USA) and 1% agarose gel electrophoresis. Next, 1μg of properly purified RNA (OD 260/280 between 1.8 and 2.1 and intact rRNA subunits - 28S and 18S) was used for cDNA synthesis by using the Maxima First Strand cDNA Synthesis kit for RT-qPCR with dsDNase (Thermo Scientific^®^, USA). A negative control RT reaction (without reverse transcriptase enzyme) was prepared for each sample.

## Primer design and efficiency estimation for qPCR

To determine the T-helper response (Th1/Th2/Th17/Th22), primers were used for the following targets: STAT4, JAK2, T-bet, STAT6, GATA3, JAK3, STAT3, RORγt, and FOXO4. Inflammasome activation profile was assessed by using primers for the following targets: Dectin1, NLRP3, Caspase1, MyD88, IL6, and IL18. Exhaustion profile was evaluated by using primers for the following targets: PRDM-1 and HAVCR-2. RT-qPCR reaction was performed with a final volume of 10µL by using the Fast SYBR^®^ Green Master Mix kit (Applied Biosystems^®^, USA), and the AriaMx Real-Time PCR system (Agilent Technologies^®^, USA) was used for reading. The reference genes, B2N and GAPDH, were used for relative quantification of all targets.

## Statistical analysis

Statistical significance was determined by using Mann-Whitney U-test for comparisons between two groups and Kruskal-Wallis test, followed by Dunn’s multiple comparisons for multi-group analyses. Statistical significance was set at p ≤ 0.05.

## RESULTS

### Study population

This is a retrospective clinical observational study to evaluate the association between several clinical/laboratory findings and comorbidities in a cohort of unvaccinated patients hospitalized in the ward or intensive care unit (ICU) in Recife, Pernambuco State, Brazil, during the beginning of the COVID-19 pandemic. A total of 36 participants (19 females [52.8%] and 17 males [47.2%]) with a mean age of 57±14 years were evaluated, of which 58.3% were hospitalized in the ward and 41.7% in ICU due to COVID-19 severity, with an estimated hospitalization of 9.31 ± 8.40 days. The criterion for hospitalization in the ICU was the development of severe acute respiratory distress syndrome (ARDS), which required invasive mechanical ventilation. All the patients had coughs, fever, and dyspnea as the main symptoms. However, depending on the patient group, the prevalence of other symptoms was observed (Supplementary Table S1). Furthermore, patients with comorbidities had a greater need for oxygen supplementation, in which 21.43% had no comorbidity (G1), 87.5% had arterial hypertension (G2), 50% had hypertension associated with diabetes (G3), and 50% had metabolic syndrome (G4).

### Impact of comorbidities on clinical laboratory findings

Depending on the clinical suspicion, cultures were made from blood samples, urine, tracheal/pleural secretion, nasal swabs, and rectal swabs to investigate the presence of bacterial co-infections. Results showed that blood and urine cultures were the most prevalent laboratory tests in the groups, except for G4 (Table 1). Supplementary Tables S1 and S2 detail, respectively, that medication therapy was directly related to specific symptoms of the patients in each experimental group. Additionally, the treatment choice was based on the microbial co-infection profile identified in the microbiological examinations.

**Table 1:** Distribution of bacterial species and their antibiotic resistance profiles in clinical samples.

Group	Culture source	Exams performed		Bacterial species	Antibiotics resistance
G1	Blood culture	40%	(+) 33.3% (-) 66.7%	*Pseudomonas putida*	Ceftriaxone, Trimethoprim-sulfamethoxazole.
	Urine culture	40%	(+) 16.7% (-) 83.3%	*Escherichia coli*	Cefepime
	Tracheal and pleural secretion	13,29%	(+) 100% (-) 0%	*Klebsiella pneumoniae*	Ceftazidime, Tigecycline.
	Rectal swab	33.3%	(+) 20% (-) 80%	*Acinetobacter baumannii, Stenotrophomonas maltophilia*	Meropenem, Ceftazidie, Imipenem.
	Nasal swab	20%	(+) 50% (-) 50%	*Staphylococcus haemolyticus*	Chloramphenicol, Gentamicin, Tetracycline.
G2	Blood culture	42,9%	(+) 66.7% (-) 33.3%	*Staphylococcus epidermidis, Klebsiella pneumoniae*	Tigecycline, Ciprofloxacin.
	Urine culture	28,6%	(+) 100% (-) 0%	*Klebsiella pneumoniae*	Ciprofloxacin, Trimethoprim-sulfamethoxazole.
	Tracheal and pleural secretion	14,3%	(+) 100% (-) 0%	*Achromobacter* spp.	Amikacin, Gentamicin.
	Rectal swab	28.6%	(+) 100% (-) 0.0%	*Klebsiella pneumoniae, Acinetobacter baumannii*	Levofloxacin, Tigecycline.
	Nasal swab	14.3%	(+) 100% (-) 0%	*Klebsiella pneumoniae*	Imipenem, Levofloxacin.
G3	Blood culture	72.4%	(+) 40% (-) 60%	*Staphylococcus epidermidis, Staphylococcus capitis.*	Levofloxacin, Trimethoprim-sulfamethoxazole, Teicoplanin.
	Urine culture	85.7%	(+) 33.3% (-) 66.7%	*Enterococcus faecalis*	Gentamicin
	Tracheal and pleural secretion	57.1%	(+) 83% (-) 16.7%	*Klebsiella pneumoniae, Proteus mirabilis, Pseudomonas aeruginosa.*	Amikacin, Cefepime, Tigecycline.
	Rectal swab	57.1%	(+) 25% (-) 75%	*Stenotrophomonas maltophilia*	Imipenem, Meropenem.
G4	Blood culture	42.9%	(+) 66.7% (-) 33.3%	*Staphylococcus haemolyticus, Staphylococcus epidermidis*	Rifampicin, Teicoplanin, Ciprofloxacin
	Urine culture	42.9%	(+) 66.7% (-) 33.3%	*Pseudomonas aeruginosa, Klebsiella pneumoniae, Enterococcus faecalis*	Gentamicin, Streptomycin, Levofloxacin
	Tracheal and pleural secretion	42.9%	(+) 100% (-) 0%	*Acinetobacter baumannii, Staphylococcus haemolyticus, Enterococcus faecalis*	Levofloxacin, Gentamicin
	Rectal swab	57.1%	(+) 25% (-) 75%	*Pseudomonas aeruginosa*	Amikacin, Imipenem, Meropenem
	Nasal swab	42.9%	(+) 66.7% (-) 33.3%	*Staphylococcus haemolyticus, Acinetobacter baumannii*	Levofloxacin, Amikacin

**Figure 2:**
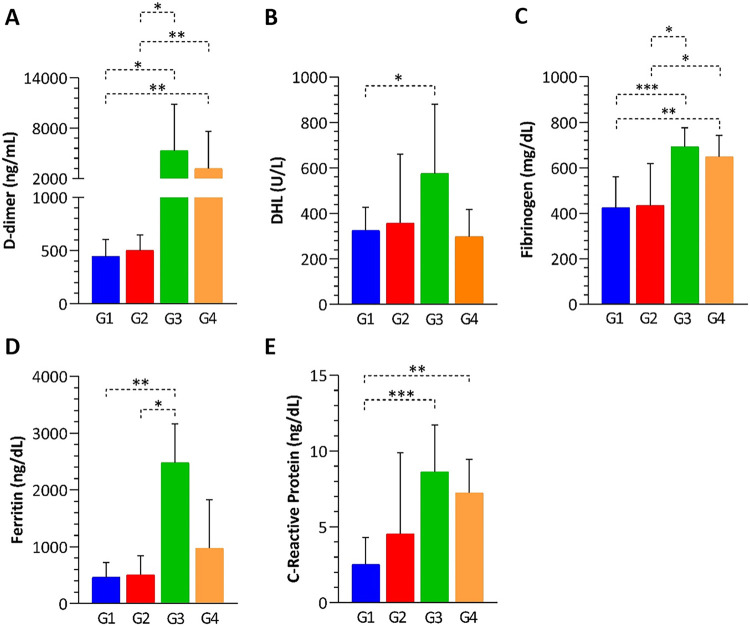
Biochemical markers found in COVID-19 patients: (A) D-dimer; (B) LACTATE dehydrogenase (LDH); (C) Fibrinogen; (D) Ferritin; (E) C-reactive protein (CRP). G1: patients without comorbidities; G2: patients with systemic arterial hypertension (SAH); G3: patients with systemic arterial hypertension and diabetes mellitus; G4: patients with metabolic syndrome (MetS). Statistical analyses were performed using the non-parametric Mann-Whitney test. *p*-values are denoted as * for *p* < 0.05, ** for *p* < 0.01, and *** for *p* < 0.001.

Figure 2 shows that biochemical analyses showed significant differences between the groups. Patients with hypertension associated with diabetes (G3) had great differences in all biochemical markers. In contrast, the group with metabolic syndrome (G4) showed elevated levels of D-dimer, fibrinogen, and C-reactive protein. Patients with hypertension (G2) also had a profile like those without comorbidities (G1). Leukocytosis, neutrophilia, and lymphopenia were more pronounced in G3 and G4. Supplementary Figure 1 showed the hematological findings.

### Effect of comorbidities on immunological parameters in distinct ways

**Figure 3:**
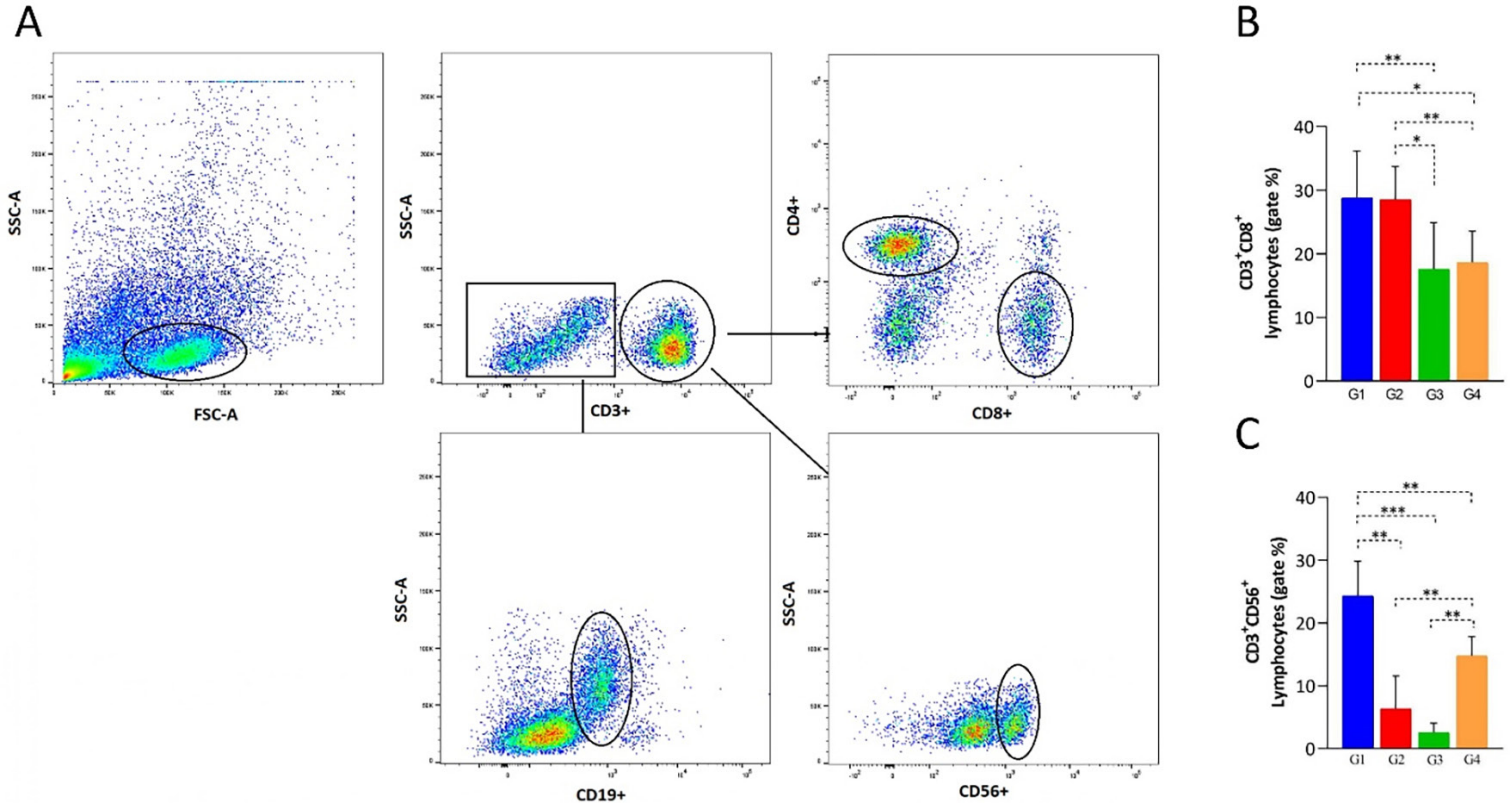
- Lymphocyte immunophenotyping in patients with COVID-19: (A) flow cytometry gating strategy used to define populations of T and B lymphocytes, and NK cells; (B and C) results of CD8 T lymphocytes (CD8a+) and NK cells (CD56+), showing reductions in both populations in groups G3 and G4. G1: patients without comorbidities; G2: patients with systemic arterial hypertension (SAH); G3: patients with systemic arterial hypertension and diabetes mellitus; G4: patients with metabolic syndrome (MetS). Statistical analyses were performed using the non-parametric Mann-Whitney test. *p*-values are denoted as * for *p* < 0.05, ** for *p* < 0.01, and *** for *p* < 0.001.

**Figure 4:**
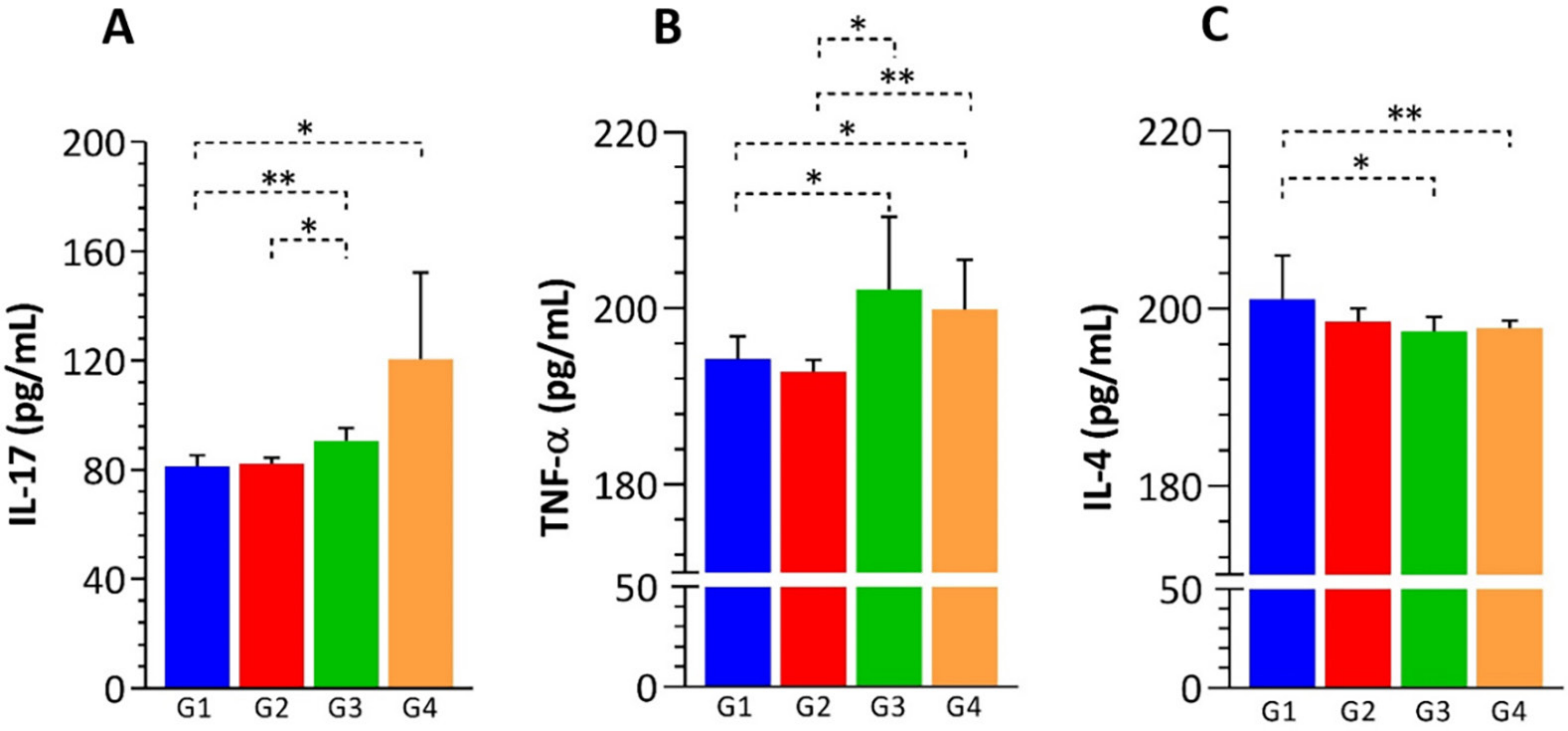
Cytokine profile in the serum of COVID-19 patients in different clinical contexts: (A) IL-17 levels; (B) TNF-alpha levels; (C) IL-4 levels. G1: patients without comorbidities; G2: patients with systemic arterial hypertension (SAH); G3: patients with systemic arterial hypertension and diabetes mellitus; G4: patients with metabolic syndrome (MetS). Statistical analyses were performed using the non-parametric Mann-Whitney test. *p*-values are denoted as * for *p* < 0.05, ** for *p* < 0.01, and *** for *p* < 0.001.

Differential counts in the immunophenotyping showed variations in lymphocyte populations (Figure 3). CD8^+^ T lymphocytes were highly reduced in patients with hypertension associated with diabetes (G3) and metabolic syndrome (G4). G2, G3, and G4 also showed a reduction in the NK (CD56^+^) lymphocytes. No significant differences were observed in T CD4^+^ and B (CD19^+^) lymphocytes, and the cytokine analysis revealed increased levels of IL-17 and TNF-α, along with reduced levels of IL-4 in G3 and G4, when compared with those of the other groups (Figure 4).

**Figure 5:**
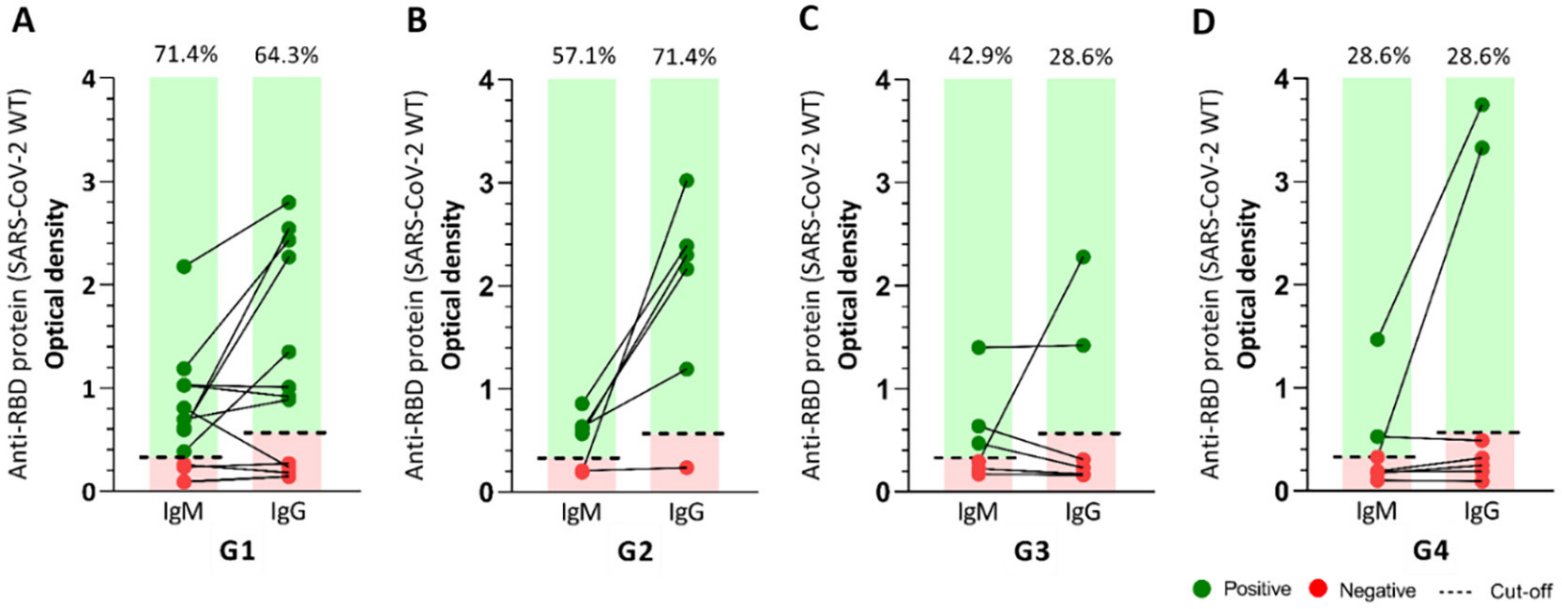
Seroconversion of IgM and IgG in the groups studied: G1: patients without comorbidities; G2: patients with systemic arterial hypertension (SAH); G3: patients with systemic arterial hypertension and diabetes mellitus; G4: patients with metabolic syndrome (MetS).

The seroconversion rates of IgM and IgG specific to Wuhan SARS-CoV-2 RBD were analyzed in all groups. Although all patients were diagnosed with COVID-19, the IgM seroconversion rate decreased in the presence of comorbidities, which was four times lower in G4 compared to G1 (patients without comorbidities). Figure 5 shows that the seroconversion rate in patients of G3 and G4 was similar for IgG, and was at least three times lower than G1 and G2. Neutralization assays were performed by using the same samples against the Wuhan, P1/Gamma, Delta, and Omicron SARS-CoV-2 variants, and no sample showed antibodies neutralizing any of the SARS-CoV-2 variants tested. Notably, despite the differences in immune response, the mean number of hospitalization days prior to sample collection was comparable between the groups, namely: G1: 13.5 ± 6.6 days, G2: 18.0 ± 6.5 days, G3: 24.8 ± 7.3 days, and G4: 14.0 ± 4.2 days.

Gene expression analysis revealed distinct differences between the groups regarding STAT4/T-bet and STAT3/RORγt markers, which are associated with specific profiles of T-helper cells (Th1 and Th17). G3 and G4 showed significantly higher expression of STAT3 compared to STAT4 and higher expression of RORγt compared to T-bet, indicating a predominant Th17 profile. In contrast, G2 showed no statistical difference between STAT3 and STAT4 expression, but there was an elevated T-bet expression, suggesting a Th1 profile. Supplementary Figure S2 shows the relative expression profile.

Our findings revealed that G3 showed a significant increase in the expression of NLRP3 and IL-18 genes, when compared with the other groups. Additionally, G3 and G4 showed high levels of Dectin-1 expression when compared with the other groups. Moreover, patients with metabolic syndrome (G4) also showed elevated levels of MyD88 expression (Supplementary Figure S3).

The gene expression profiles associated with lymphocyte exhaustion were analyzed, focusing on the markers HAVCR-2 and PRDM-1. Supplementary Figure S4 shows statistically significant differences in the expression levels of the HAVCR-2 gene between the groups. However, PRDM-1 showed no statistically significant differences.

## DISCUSSION

We evaluated the impact of different comorbidities on clinical outcomes, laboratory findings, and immune profile of unvaccinated patients with COVID-19. Firstly, we investigated their importance in the prevalence of clinical symptoms. Our findings showed that hypertension (G2) was the most common comorbidity associated with the need for oxygen supplementation, followed by a combination of hypertension and diabetes (G3) and metabolic syndrome (G4). Several studies had similar results, in which hypertension was associated with more severe lung injury, frequent need for ICU admission, increased necessity of invasive ventilation, and consequently, higher mortality^16-19^. Uncontrolled hypertension presumedly causes several pathophysiological changes in the heart and blood vessels, including endothelial dysfunction, left ventricular hypertrophy and fibrosis, which may increase the myocardium’s and endothelium’s susceptibility to SARS-CoV-2 infection^20^.

Secondly, the clinical outcomes highlight the significant impact of comorbidities on biochemical and hematological parameters. Patients with hypertension and diabetes (G3) showed the most pronounced alterations, with significant changes in all key biochemical markers, which suggests a more severe systemic inflammatory response. In contrast, patients with isolated hypertension (G2) showed more biochemical and hematological profiles comparable to patients without comorbidities (G1), which suggests a milder clinical course. Furthermore, leukocytosis, neutrophilia, and lymphopenia were notably more severe in G3 and G4, thus reinforcing that multiple or more complex comorbidities exacerbate the immunological and clinical responses to the underlying condition.

Thirdly, knowing that co-infections and pre-existing medical conditions can significantly impact on how the organism responds to the SARS-CoV-2 virus and often lead to more complicated clinical courses, we evaluated the presence of bacterial species, their respective antibiotic resistance profiles in the samples and the medication frequency in each group. *Staphylococcus epidermidis*, *Acinetobacter baumannii*, and *Klebsiella pneumoniae* were prevalent in our results. Other studies have also found *Streptococcus pneumoniae*, *Staphylococcus* species, *Klebsiella pneumoniae*, and *Acinetobacter baumannii* to be the most prevalent highly-resistant microorganisms in COVID-19 patients^21-23^. Additionally, the use of invasive devices, such as nasal catheters and orotracheal intubation, along with reduced immune function may be potential reasons for a higher rate of nosocomial infection in COVID-19 patients^24^.

Moreover, the medications currently used in the management of COVID-19, while not specifically targeting the SARS-CoV-2 virus, can lead to other complications such as the hepatotoxic and nephrotoxic effects associated with certain corticosteroids and antimicrobials^25^. Since some patients already had comorbidities (e.g. diabetes and hypertension), the side effects of these drugs may have been exacerbated, potentially leading to the worsening of these pre-existing conditions. This reasoning is associated with the observational analysis of some thrombotic and coagulative complications that patients began to show in the post-acute phase of COVID-19. The indiscriminate use of antibiotics (e.g. ceftriaxone and azithromycin) to treat COVID-19 implicitly increases the rate of bacterial resistance^26,27^. In this case, the need of greater care it is inferred in clinical practice regarding the predominant choice of beta-lactamase-resistant and broad-spectrum drugs, such as carbapenems (e.g. imipenem and meropenem) as well as third and fourth generation cephalosporins^28^.

Next, we aimed to evaluate the influence of comorbidities in biochemical and hematological parameters. We observed that the groups with hypertension associated with diabetes (G3) and with metabolic syndrome (G4) showed significantly elevated levels of D-dimer, fibrinogen, and C-reactive protein, as well as pronounced leukocytosis with neutrophilia and lymphopenia, when compared with the other groups. Patients with comorbidities such as diabetes and metabolic syndrome already have chronic inflammation, which increases the risk of severe types of COVID-19, including severe acute respiratory syndrome (SARS)^29,30^. During the pandemic, inflammatory biomarkers (e.g. D-dimer and ferritin) rose even further, exacerbating inflammation and tissue damage^31^. This pre-existing inflammation, combined with the inflammatory response to the virus, worsens endothelial dysfunction and activates the coagulation system, which worsens the severity of SARS and the prognosis^32^. Neutrophilia and reduced lymphocyte counts, observed in COVID-19 patients, are particularly amplified in individuals with diabetes, negatively affecting peripheral blood immune cells^33^. Such findings together reveal how diabetes and glycemic disorders can influence the severity and prognosis of the disease.

Serum levels of antiviral antibodies were measured in the samples to understand the patient’s humoral immunity. We observed that the seroconversion rate in G3 and G4 was very similar, being at least three times lower than that of the other groups. This reduced humoral immunity in patients with COVID-19 in the pre-vaccination period is characterized by a less efficient seroconversion and low neutralizing capacity, which may be directly related to pre-existing inflammatory comorbidities, thus impacting the effectiveness of the adaptive immune response and possibly affecting the function of B lymphocytes^34^. Therefore, these patients pose significant challenges in building an effective immune response to COVID-19 infection, making them more susceptible to severe forms of the disease.

In general, unvaccinated patients face greater difficulty in neutralizing new variants of SARS-CoV-2 due to the lack of immune memory and immune evasion mechanisms of these variants. Moreover, comorbidities further limit the ability to generate an effective immune response within our cohort, especially to develop efficient neutralizing antibodies. Some studies also indicate that a large proportion of unvaccinated individuals showed only low or no neutralizing activity against the virus at the beginning of infections by the Ômicron-BA.2 variant, which suggests that in addition to the inflammatory status, the immune response in unvaccinated individuals may be less robust and less effective than the vaccinated ones^35^.

Uncontrolled immune responses of T-cells during COVID-19 have been strongly linked to severe outcomes, tissue damage, and organ failure underscoring the importance of understanding T cell dynamics in this disease^36^. Other studies also suggest that the predominant immune response in severe COVID-19 often involves excessive activation of type 1 helper T cells (Th1), followed by type 17 helper T cells (Th17). In this context, the up-regulation of transcription factors associated with Th1 and Th17 implies an exacerbated pro-inflammatory response, specifically in COVID-19 patients, which leads to cytokine production such as IFN-γ, IL-17, and IL-6^37^.

However, we found that the predominance of Th17 response in patients with multiple comorbidities (G3 and G4) is likely associated with the immune status of the patients, which is possibly linked to insulin resistance^35^. The cytokine IL-17 is also implicated in inflammation, insulin resistance, and development of type 2 diabetes (T2D). Consistent with our findings, patients with poor glycemic control showed elevated levels of IL-17 and other inflammatory cytokines, such as interferon-gamma (IFN-γ), indicating a role for Th17 cells in the pathophysiology of diabetes^38^. The pre-existing Th17 response in diabetic patients may strongly indicate exacerbation when combined with inflammation induced by COVID-19^37,38^.

The activation of inflammasome, especially NLRP3, in chronically inflamed patients, such as those with type 2 diabetes, is associated with the production of pro-inflammatory cytokines (e.g., IL-1β and IL-18)^39^. This process promotes a cycle of exacerbated inflammation, resulting in tissue damage, organ dysfunction, and severe complications, such as acute respiratory distress syndrome (ARDS) and multi-organs failure. The interaction between COVID-19 and comorbidities (e.g., diabetes) can improve the inflammatory response, increasing the expression of inflammatory markers such as NLRP3, ASC and caspase-1, thus elevating the risk of severe complications^39^. Our findings are aligned with previous research in which diabetic patients showed increased levels of markers such as NLRP3 and IL-18^40^. The increased expression of the MyD88 marker is likely a reflection of obesity, particularly in the context of metabolic syndrome. Such elevated MyD88 signaling may predispose obese individuals to develop an exaggerated and potentially dangerous inflammatory response to SARS-CoV-2 infection^40^.

### Study limitations

The sample size was not representative of the city’s population, but rather of the number of COVID-19 patients hospitalized during the study period. The Clinic Hospital of the Federal University of Pernambuco is small to medium sized, and only 610 patients were admitted during the study period, of which 90 were diagnosed with COVID-19 and only 36 had some comorbidity. Additionally, we did not establish a comparison with vaccinated patients due to the variety of doses and types of vaccines administered to the Brazilian population.

## CONCLUSION

This study evaluated the impact of comorbidities on the clinical profile, immune response, and laboratory findings of unvaccinated COVID-19 patients. We found that hypertension, diabetes, and metabolic syndrome significantly worsened the disease severity, and patients in these groups required more oxygen supplementation and had elevated inflammatory markers. Bacterial co-infections by *Staphylococcus epidermidis* and *Klebsiella pneumoniae* were common, thus highlighting the importance of monitoring antibiotic resistance. Although necessary, COVID-19 treatments with corticosteroids and antimicrobials exacerbated existing conditions and worsened the outcomes. Although all patients developed IgG antibodies, none had a neutralizing response, which highlights the importance of the cellular response in COVID-19. Furthermore, immune dysregulation and chronic inflammation, particularly in diabetic patients, contributed to severe complications. Considering these findings and the distinct behavior of this group of patients, we highlight the need for careful management of comorbidities and reinforce the need for further studies on the interaction between diabetes and COVID-19.

## References

[1] Centers for Disease Control and Prevention People with certain medical conditions and COVID-19 risk factors.

[2] Centers for Disease Control and Prevention Underlying conditions and the higher risk for severe COVID-19.

[3] Malireddi RK, Sharma BR, Kanneganti TD (2024). Innate immunity in protection and pathogenesis during coronavirus infections and COVID-19. Annu Rev Immunol.

[4] Lundstrom K, Hromic-Jahjefendic A, Bilajac E, Aljabali AA, Baralic K, Sabri NA (2023). COVID-19 signalome: pathways for SARS-CoV-2 infection and impact on COVID-19 associated comorbidity. Cell Signal.

[5] Moreira RS, Costa EG, Santos LF, Miranda LH, Oliveira RR, Romão RF (2022). The assistance gaps in combating COVID-19 in Brazil: for whom, where and when vaccination occurs. BMC Infect Dis.

[6] Evered JA, Castellanos ME, Dowrick A, Germani AC, Rai T, Souza AN (2023). Talking about inequities: a comparative analysis of COVID-19 narratives in the UK, US, and Brazil. SSM Qual Res Health.

[7] Martines MR, Ferreira RV, Toppa RH, Assunção LM, Desjardins MR, Delmelle EM (2021). Detecting space-time clusters of COVID-19 in Brazil: mortality, inequality, socioeconomic vulnerability, and the relative risk of the disease in Brazilian municipalities. J Geogr Syst.

[8] Oliveira M, Braga MF, Bueno A, Sousa DP, Pigozi PL, Moryia R (2022). Actions during the COVID-19 pandemic to protect the most vulnerable population: what is the potency amid chaos?. Health Promot Int.

[9] Sociedade Brasileira de Diabetes Brasil já tem cerca de 20 milhões de pessoas com diabetes.

[10] Hospital Alemão Osvaldo Cruz Hipertensão atinge 30% da população adulta brasileira.

[11] Expert Panel on Detection, Evaluation and Treatment of High Blood Cholesterol in Adults (2001). Executive summary of the Third Report of the National Cholesterol Education Program (NCEP) Expert Panel on Detection, Evaluation, and Treatment of High Blood Cholesterol in Adults (Adult Treatment Panel III). JAMA.

[12] Clinical and Laboratory Standard Institute (2020). Performance standards for antimicrobial susceptibility testing.

[13] Venceslau-Carvalho AA, Favaro MT, Pereira LR, Rodrigues-Jesus MJ, Pereira SS, Andreata-Santos R (2021). Nano-multilamellar lipid vesicles loaded with a recombinant form of the chikungunya virus E2 protein improve the induction of virus-neutralizing antibodies. Nanomedicine.

[14] Souza MS, Farias JP, Andreata-Santos R, Brito RD, Souza MS, Fogaça MM (2024). Neutralizing antibody response after immunization with a COVID-19 bivalent vaccine: insights to the future. J Med Virol.

[15] Pires FJ, Andreata-Santos R, Dalety SB, Silva SM, Moreira CF, Ramos PJ (2023). The fourth COVID-19 vaccine dose increased the neutralizing antibody response against the SARS-CoV-2 Omicron (B.1.1.529) variant in a diverse Brazilian population. Microbiol Spectr.

[16] Podzolkov VI, Bragina AE, Tarzimanova AI, Vasilyeva LV, Ogibenina ES, Bykova EE (2023). Arterial hypertension and severe COVID-19 in hospitalized patients: data from a cohort study. Ration Pharmacother Cardiol.

[17] Peng M, He J, Xue Y, Yang X, Liu S, Gong Z (2021). Role of hypertension on the severity of COVID-19: a review. J Cardiovasc Pharmacol.

[18] Kreutz R, Algharably EA, Azizi M, Dobrowolski P, Guzik T, Januszewicz A (2020). Hypertension, the renin-angiotensin system, and the risk of lower respiratory tract infections and lung injury: implications for COVID-19. Cardiovasc Res.

[19] Du Y, Zhou N, Zha W, Lv Y (2021). Hypertension is a clinically important risk factor for critical illness and mortality in COVID-19: a meta-analysis. Nutr Metab Cardiovasc Dis.

[20] Ambrosino P, Bachetti T, D'Anna SE, Galloway B, Bianco A, D'Agnano V (2022). Mechanisms and clinical implications of endothelial dysfunction in arterial hypertension. J Cardiovasc Dev Dis.

[21] Fattorini L, Creti R, Palma C, Pantosti A (2020). Bacterial coinfections in COVID-19: an underestimated adversary. Ann Ist Super Sanita.

[22] Dumitru IM, Dumitrascu M, Vlad ND, Cernat RC, Ilie-Serban C, Hangan A (2021). Carbapenem-resistant Klebsiella pneumoniae associated with COVID-19. Antibiotics (Basel).

[23] Raoofi S, Kan FP, Rafiei S, Hosseinipalangi Z, Mejareh ZN, Khani S (2023). Global prevalence of nosocomial infection: a systematic review and meta-analysis. PloS One.

[24] Ong CC, Farhanah S, Linn KZ, Tang YW, Poon CY, Lim AY (2021). Nosocomial infections among COVID-19 patients: an analysis of intensive care unit surveillance data. Antimicrob Resist Infect Control.

[25] Movahed SM, Akhavizadegan H, Dolatkhani F, Nejadghaderi SA, Aghajani F, Gangi MF (2021). Different incidences of acute kidney injury (AKI) and outcomes in COVID-19 patients with and without non-azithromycin antibiotics: a retrospective study. J Med Virol.

[26] Ruiz J (2021). Enhanced antibiotic resistance as a collateral COVID-19 pandemic effect?. J Hosp Infect.

[27] Sharifipour E, Shams S, Esmkhani M, Khodadadi J, Fotouhi-Ardakani R, Koohpaei A (2020). Evaluation of bacterial co-infections of the respiratory tract in COVID-19 patients admitted to ICU. BMC Infect Dis.

[28] Bahçe YG, Acer Ö, Özüdogru O (2022). Evaluation of bacterial agents isolated from endotracheal aspirate cultures of Covid-19 general intensive care patients and their antibiotic resistance profiles compared to pre-pandemic conditions. Microb Pathog.

[29] Hussain A, Bhowmik B, Moreira NC (2020). COVID-19 and diabetes: knowledge in progress. Diabetes Res Clin Pract.

[30] Russell CD, Lone NI, Baillie JK (2023). Comorbidities, multimorbidity and COVID-19. Nat Med.

[31] Ali HN, Ali KM, Rostam HM, Ali AM, Tawfeeq HM, Fatah MH (2022). Clinical laboratory parameters and comorbidities associated with severity of coronavirus disease 2019 (COVID-19) in Kurdistan Region of Iraq. Pract Lab Med.

[32] Tang N, Li D, Wang X, Sun Z (2020). Abnormal coagulation parameters are associated with poor prognosis in patients with novel coronavirus pneumonia. J Thromb Haemost.

[33] Prasad M, Chen EW, Toh SA, Gascoigne NR (2020). Autoimmune responses and inflammation in type 2 diabetes. J Leukoc Biol.

[34] Cunha LL, Perazzio SF, Azzi J, Cravedi P, Riella LV (2020). Remodeling of the immune response with aging: immunosenescence and its potential impact on COVID-19 immune response. Front Immunol.

[35] Zhang H, Jiang Y, Tan H, Zou L, Zheng Z, Huang Y (2022). Assessment of antibody responses against SARS-CoV-2 in unvaccinated individuals and vaccinees from Omicron-BA.2 infection in Zhaoqing, Guangdong Province, China. Virol J.

[36] Abbasi-Dokht T, Vafaeinezhad A, Khalesi N, Malek F, Haghmorad D, Baharlou R (2023). T-cell immune responses and immunological factors associated with coronavirus disease 2019 progression as predictors for the severity of the disease in hospitalized patients. Int Arch Allergy Immunol.

[37] Jovanovic M, Sekulic S, Jocic M, Jurisevic M, Gajovic N, Jovanovic M (2023). Increased pro Th1 and Th17 transcriptional activity in patients with severe COVID-19. Int J Med Sci.

[38] Gil-Etayo FJ, Suàrez-Fernández P, Cabrera-Marante O, Arroyo D, Garcinuño S, Naranjo L (2021). T-helper cell subset response is a determining factor in COVID-19 progression. Front Cell Infect Microbiol.

[39] Kang YW, Lee SC, Jeon SM, Jo EK (2021). Roles of Interleukin-17 and Th17 responses in COVID-19. J Bacteriol Virol.

[40] Lu S, Li Y, Qian Z, Zhao T, Feng Z, Weng X (2023). Role of the inflammasome in insulin resistance and type 2 diabetes mellitus. Front Immunol.

